# Non-signaling protein therapeutics: a mechanism-based regulatory framework to modernize biosimilar development and global harmonization

**DOI:** 10.3389/fmed.2026.1779934

**Published:** 2026-03-12

**Authors:** Sarfaraz K. Niazi

**Affiliations:** College of Pharmacy, University of Illinois, Chicago, IL, United States

**Keywords:** analytical similarity, biosimilars, complement inhibition, functional characterization, interchangeability, ligand neutralization, mechanism-based classification, non-signaling proteins

## Abstract

Non-Signaling Protein Therapeutics (NSPTs) represent a proposed regulatory category for biological products whose therapeutic effects operate through ligand neutralization, complement cascade blockade, or other non-signal-transducing mechanisms rather than receptor-mediated signaling. These products exhibit bounded biological responses, with a direct relationship between measurable functional activity and clinical effects, making analytical and functional characterization inherently predictive of therapeutic performance. Analysis of FDA-licensed biologics identifies 43 products that meet NSPT criteria, including anti-TNF, anti-VEGF, anti-interleukin, and complement-inhibitor therapeutics. While some NSPTs have robust biosimilar competition, many high-value products lack biosimilar development despite patent expiration, creating a development gap that may affect resource allocation and patient access timelines. A comprehensive timeline documenting regulatory evolution from BPCIA enactment in 2010 through FDA draft guidance proposing elimination of default clinical efficacy study requirements illustrates a systematic progression toward evidence-efficient frameworks. The NSPT framework proposes fit-for-purpose evidence packages prioritizing comprehensive analytical characterization and mechanistic potency assays, reserving clinical studies for specific residual uncertainties. This approach aligns with FDA modernization initiatives and historical precedents demonstrating regulatory flexibility. Implementation could potentially reduce clinical trial requirements that may not add commensurate scientific value, while maintaining safety and efficacy standards. The framework proposes that NSPT biosimilars meeting analytical and functional criteria, recognizing that mechanism-based similarity supports therapeutic equivalence, may warrant consideration for interchangeability designation, subject to statutory requirements. Regulatory agencies should consider establishing NSPT classification guidelines, harmonizing international standards, and evaluating whether current evidence requirements are optimally calibrated to scientific uncertainty for these product classes.

## 1 Introduction

The landscape of biosimilar development stands at a critical juncture where scientific advancement, regulatory evolution, and policy considerations converge, prompting evaluation of the evidence required to demonstrate biosimilarity. The evolution of biosimilar regulatory science has reached an inflection point at which mechanism-based classification may substantially reduce the development burden without compromising safety or efficacy standards, while addressing important considerations regarding resource allocation and patient access inherent in current regulatory approaches. This review introduces and operationalizes Non-Signaling Protein Therapeutics (NSPTs), a proposed regulatory category encompassing biological products (1) whose primary therapeutic effects are mediated by ligand neutralization, complement cascade blockade, or other non-signal-transducing biochemical interception mechanisms rather than by receptor-mediated signal transduction.

The timing for this classification is particularly opportune given the FDA’s November 2025 draft guidance explicitly focusing on assessing when comparative clinical efficacy studies are necessary for biosimilar approval, marking a fundamental shift from treating such studies as default requirements to conditional tools deployed only when residual uncertainty persists after robust analytical and functional characterization (2, 3). This regulatory evolution reflects growing recognition that requiring extensive clinical trials for products with predictable, mechanism-based pharmacology may represent suboptimal resource allocation—potentially consuming limited clinical research resources, delaying patient access to affordable treatments, and contributing to healthcare cost challenges that warrant careful consideration.

The resource implications of biosimilar clinical trials merit careful consideration. Clinical trials that confirm results already demonstrated through analytical science enroll hundreds or thousands of patients, potentially diverting participation from studies investigating novel therapeutics. Healthcare systems managing biological drug expenditures may face extended timelines before biosimilar competition, as sponsors conduct trials whose incremental informational value warrants scrutiny. These considerations regarding human, financial, and temporal resource allocation support the evaluation of whether current evidence requirements are optimally calibrated to scientific uncertainty.

This comprehensive analysis demonstrates that NSPTs exhibit bounded biological nonlinearity and maintain direct relationships between measurable functional potency and clinical effects, making well-validated functional assay panels intrinsically predictive of therapeutic performance. Evidence from multiple therapeutic areas shows that ligand-neutralizing antibodies targeting tumor necrosis factor alpha, vascular endothelial growth factor, receptor activator of nuclear factor kappa-B ligand, and interleukin families consistently demonstrate predictable dose–response relationships that can be fully characterized through orthogonal binding and neutralization assays. Similarly, complement-blocking therapeutics such as eculizumab and rovelizumab show a direct correlation between complement inhibition in validated hemolysis assays and clinical outcomes across all approved indications.

Our systematic assessment of currently FDA-licensed biological products identifies 43 reference biologics that meet NSPT criteria across inflammatory, ophthalmologic, metabolic, neurologic, and hematologic therapeutic areas, based on Purple Book data through November 2025 (1, 4). Products were identified through systematic screening using the following protocol: the data source was the FDA Purple Book database; inclusion criteria required current licensure under BLA with primary mechanism involving ligand neutralization, complement blockade, or non-signaling interception where therapeutic effect is directly measurable via validated functional assays; exclusion criteria included receptor agonists, immune checkpoint modulators, and products with complex cell-mediated mechanisms (5). Critical analysis reveals that while some NSPT-eligible molecules have achieved robust biosimilar competition with multiple approved products, including adalimumab with ten biosimilars and bevacizumab with four, many high-value NSPTs remain in what the IQVIA Institute characterizes as the biosimilar void, with no biosimilars in late-stage development despite approaching patent expiration (6). This disparity underscores the potential benefits of mechanism-based regulatory clarity (7) in reducing development uncertainty and costs.

The traditional paradigm of defaulting to comparative clinical efficacy studies regardless of mechanism has increasingly been recognized as scientifically unnecessary for certain classes of biological products and economically prohibitive for sustainable biosimilar competition (8). This recognition has catalyzed regulatory modernization efforts, culminating in the FDA’s November 2025 draft guidance, which explicitly reframes comparative clinical efficacy studies from presumptive requirements to conditional tools used when residual uncertainty remains after analytical and functional characterization (2).

The economic stakes of this modernization cannot be overstated. The biosimilar market has generated over $36 billion in cumulative savings (9) for the United States healthcare system since the first biosimilar approval in 2015, with $12 billion in savings achieved in 2023 alone (8). Yet paradoxically (10), the IQVIA Institute identifies a substantial biosimilar void: 118 biological products approaching patent expiration through 2034, representing $232 billion in estimated spending opportunity (11) (based on IQVIA modeling assumptions), have attracted biosimilar development for only 12 molecules as of June 2024 (6). This disconnect between market opportunity and development activity reflects fundamental uncertainty about evidence requirements that varies by product mechanism, therapeutic area, and regulatory precedent—uncertainty that may contribute to extended periods of limited market competition, affect pricing dynamics, and influence the pace of patient access to biological therapies.

International harmonization opportunities emerge from examining divergent regulatory approaches, particularly when products regulated as drugs under the FDA’s Federal Food, Drug, and Cosmetic Act are treated as biosimilars by the European Medicines Agency. Historical precedents, including the regulatory transition of insulin products from drug to biologic status in March 2020 and the approval of follow-on proteins like somatropin under abbreviated pathways, demonstrate regulatory flexibility in adapting evidence requirements to scientific understanding (12, 13). The NSPT framework offers a scientifically grounded pathway for convergence that could facilitate global development programs and accelerate patient access to affordable biological therapies. (14).

By establishing clear mechanistic boundaries and evidence expectations, NSPT classification represents neither deregulation nor a compromise of standards, but rather a right-sizing of evidence requirements to match biological complexity and therapeutic mechanisms. Most critically, we propose that all NSPT biosimilars that meet analytical and functional similarity criteria may warrant consideration for interchangeability designation, recognizing that, for products with predictable, non-amplifying mechanisms, demonstrated functional equivalence ensures therapeutic equivalence. This recommendation challenges regulatory agencies to move beyond cautious incrementalism toward bold reforms that prioritize patient access and healthcare sustainability.

The scientific foundation for mechanism-based regulatory classification rests on the recognition that biological products exhibit markedly different relationships among structural attributes, functional activities, and clinical effects (15) depending on their mechanisms of action. Receptor-signaling biologics that trigger or modulate intracellular signal transduction cascades can amplify minor structural differences through complex, nonlinear networks, yielding tissue-specific and context-dependent responses. Minor variations in glycosylation patterns, binding kinetics, or conformational dynamics (16) may propagate across multiple signaling nodes, producing clinically meaningful differences in therapeutic response or safety profiles. This inherent biological amplification justifies a comprehensive clinical assessment when analytical and functional characterization cannot fully predict clinical behavior.

In contrast, a substantial subset of biological products operates through mechanisms that fundamentally differ from receptor-mediated signaling. These products, which we propose to classify as Non-Signaling Protein Therapeutics, exert their therapeutic effects through direct biochemical interception mechanisms, including ligand neutralization, complement cascade blockade, and enzymatic inhibition. The clinical effects of these molecules depend primarily on their ability to bind and sequester soluble targets or interrupt proteolytic cascades, relationships that can be directly measured through validated functional assays. This mechanistic distinction creates an opportunity for evidence-based regulatory modernization that aligns development requirements with biological complexity.

The regulatory environment has evolved to recognize these mechanistic differences. The FDA’s recent draft guidance on assessing the need for comparative clinical efficacy studies represents a paradigm shift from treating such studies as default requirements to positioning them as tools for resolving specific residual uncertainties (2). The accompanying Federal Register notice explicitly contextualizes this evolution as reflecting advances in analytical science and accumulated experience in biosimilar development across diverse product classes (3). This regulatory modernization establishes the policy foundation for a formal, mechanism-based classification that could standardize expectations for evidence and reduce development uncertainty.

The proposed NSPT classification addresses three interconnected challenges facing biosimilar development. First, it provides scientific clarity by establishing mechanistic boundaries between products that require extensive clinical assessment and those for which analytical and functional characterization suffice. Second, it offers economic predictability by defining evidence packages in advance based on the mechanism rather than through case-by-case negotiation, enabling sponsors to make informed development decisions. Third, it facilitates global harmonization by creating a common framework for regulatory convergence across jurisdictions (17) that have historically diverged in their approaches to abbreviated biological product approval pathways.

## 2 Defining non-signaling protein therapeutics: mechanistic boundaries and regulatory implications

The establishment of Non-Signaling Protein Therapeutics as a distinct regulatory category requires a precise mechanistic definition that distinguishes these products from receptor-signaling biologics while encompassing the full range of non-signaling therapeutic mechanisms. We propose that NSPTs be defined as biological products whose primary therapeutic effects are mediated through mechanisms that do not require receptor-mediated signal transduction, specifically including ligand neutralization and sequestration, complement activation blockade through prevention of proteolytic cleavage, and other biochemical interception mechanisms where therapeutic performance is directly measurable by validated functional assays.

This definition intentionally focuses on mechanism rather than molecular structure, recognizing that diverse protein architectures, including monoclonal antibodies, antibody fragments, fusion proteins, and engineered binding scaffolds (18), can all function as NSPTs if their therapeutic action involves intercepting (19) soluble mediators or blocking cascade propagation rather than triggering cellular signaling. The distinction is not whether the therapeutic protein binds its target with high affinity, as both NSPTs and receptor-signaling biologics typically exhibit nanomolar or picomolar binding constants, but rather whether that binding event initiates signal transduction cascades or prevents the target from engaging its cognate receptor or substrate.

Ligand neutralization is the largest and best-characterized category of NSPTs, encompassing monoclonal antibodies and fusion proteins that bind soluble cytokines, growth factors, and other signaling molecules to prevent their interaction with cell-surface receptors. The anti-tumor necrosis factor alpha therapeutics adalimumab, infliximab, etanercept, certolizumab pegol, and golimumab exemplify this mechanism, binding soluble and membrane-bound TNF-alpha to prevent activation of TNF receptors without themselves triggering any signaling cascade (20). Similarly, the anti-vascular endothelial growth factor agents bevacizumab, ranibizumab, and aflibercept sequester VEGF family members to prevent angiogenic signaling without directly engaging VEGF receptors (21, 22).

The therapeutic effects of ligand-neutralizing NSPTs correlate directly with their ability to reduce free ligand concentrations below levels required for receptor activation. This relationship can be quantitatively characterized through validated neutralization assays that measure inhibition of ligand-induced cellular responses. For anti-TNF therapeutics, standardized cell-based bioassays using TNF-sensitive cell lines provide direct readouts of neutralization potency that correlate with clinical anti-inflammatory effects across rheumatoid arthritis, inflammatory bowel disease, and psoriatic indications (23). The bounded nature of this pharmacology, in which maximal effect occurs when the ligand is fully neutralized, contrasts sharply with that of receptor agonists, which can trigger amplifying cascades with effects that vary by tissue context and receptor expression levels.

Complement cascade inhibitors constitute a second major category of NSPTs that function by preventing proteolytic activation of complement components rather than by engaging receptors. Eculizumab and its long-acting variant, rovelizumab, bind to complement component C5 to prevent its cleavage into C5a and C5b, thereby blocking the formation of the membrane attack complex without triggering cellular signaling pathways (24, 25). The therapeutic effect depends entirely on the degree of C5 blockade achievable at clinically relevant drug concentrations, a parameter directly measurable through hemolysis inhibition assays that correlate with clinical outcomes in paroxysmal nocturnal hemoglobinuria, atypical hemolytic uremic syndrome, and other complement-mediated disorders.

The NSPT definition explicitly excludes several categories of biologics where receptor engagement and signal transduction are fundamental to therapeutic action. Receptor agonists, including growth factors, hormones, and agonistic antibodies, are excluded because their effects depend on triggering specific signaling cascades that inherently amplify biological responses. Insulin and insulin analogs, despite their long history of abbreviated approval pathways, function as receptor agonists that initiate complex metabolic signaling and thus fall outside the NSPT framework. Cell-surface receptor antagonists that block ligand-binding sites are also excluded when their therapeutic effects depend on receptor density, internalization kinetics, or tissue-specific expression patterns that introduce biological variability beyond simple competitive inhibition.

Immune checkpoint inhibitors merit special consideration as a category that straddles the boundary between signaling and non-signaling mechanisms. While PD-1 and PD-L1 antibodies technically prevent receptor-ligand interaction like NSPTs, their therapeutic effects depend on complex immunological outcomes, including T cell activation states, tumor microenvironment composition, and systemic immune responses that extend far beyond simple ligand neutralization. The inherent complexity and context dependence of checkpoint blockade justify excluding them from the NSPT category, despite superficial mechanistic similarities to ligand neutralization.

Certain product categories warrant careful evaluation as borderline cases that illustrate the boundaries of NSPT classification. PCSK9 inhibitors, including evolocumab and alirocumab, function through straightforward ligand neutralization by binding circulating PCSK9 and preventing its interaction with hepatic LDL receptors, thereby increasing LDL receptor recycling and reducing serum cholesterol. This mechanism meets NSPT criteria as a bounded, directly measurable neutralization response. However, regulatory uncertainty regarding cardiovascular outcome study requirements has historically complicated biosimilar development for this class. The NSPT framework supports inclusion of PCSK9 inhibitors based on mechanistic principles, while acknowledging that specific regulatory expectations may evolve as experience accumulates. Similarly, certain interleukin-targeting antibodies present nuanced classification considerations. Products targeting IL-17A (secukinumab, ixekizumab) or IL-23p19 (guselkumab, tildrakizumab, risankizumab) function primarily through cytokine neutralization with bounded dose–response relationships, supporting NSPT classification. In contrast, products with broader immunomodulatory effects or those where therapeutic outcomes depend substantially on tissue-specific immune cell populations may require more extensive clinical assessment. Bispecific antibodies combining two NSPT-eligible mechanisms, such as dual cytokine neutralization, would qualify for the framework, whereas bispecific constructs engage that T cells or activate receptors would not (27). A formal dynamic classification process should be established, whereby regulatory agencies periodically review emerging therapeutic modalities against NSPT criteria, with a clear scientific rationale required for inclusion or exclusion decisions (28).

Establishing clear exclusion criteria serves both scientific and regulatory purposes. Scientifically, it preserves the mechanistic coherence of the NSPT category by limiting it to products for which functional assays provide reliable surrogates of clinical effect. Regulatory, it preserves confidence by avoiding overreach into therapeutic areas where biological complexity genuinely requires clinical assessment. This conservative approach to boundary-setting strengthens the argument for streamlined development within the NSPT category by ensuring that only products with predictable, mechanism-driven pharmacology qualify for reduced clinical evidence requirements.

## 3 Regulatory foundations: aligning NSPT classification with FDA modernization initiatives

The conceptual framework for Non-Signaling Protein Therapeutics aligns directly with ongoing FDA modernization efforts that increasingly recognize mechanism-based differences in evidence requirements for biosimilar approval. The November 2025 draft guidance on scientific considerations for assessing the need for comparative clinical efficacy studies represents a watershed moment in regulatory evolution, explicitly shifting the burden of justification from explaining why clinical studies are unnecessary to demonstrating why they are needed (2). This fundamental reorientation creates the regulatory space for formal, mechanism-based classification that could standardize expectations for evidence for products with predictable pharmacology.

### 3.1 Biosimilar regulatory history

The regulatory framework for biosimilar development has undergone significant transformation since its inception (29), reflecting the accumulation of scientific evidence and practical experience. The evolution from prescriptive, trial-intensive paradigms to analytics-first, evidence-efficient frameworks represents a fundamental shift in regulatory philosophy with profound implications for developers, regulators, and global patient access (30, 31).

This comprehensive review traces the trajectory of FDA biosimilar regulations from the passage of the Biologics Price Competition and Innovation Act (BPCIA) in 2010 through the landmark regulatory changes of 2025, documenting how accumulated scientific evidence and advocacy have systematically dismantled unnecessary testing requirements that historically impeded patient access to affordable biological medicines (Table 1).

**Table 1:** Timeline of major FDA biosimilar regulatory milestones.

Year	Regulatory milestone	Reference
2010	BPCIA, enacted as part of the Affordable Care Act, establishes the 351(k) pathway for biosimilar approval	Public Law 111–148; https://www.fda.gov/drugs/guidance-compliance-regulatory-information/implementation-biologics-price-competition-and-innovation-act-2009
2012	FDA releases draft guidance on biosimilar scientific considerations; EMA publishes mAb biosimilar guidelines	Federal Register 77 FR 8883 (Feb 15, 2012); Docket FDA-2011-D-0605; https://www.federalregister.gov/documents/2012/02/15/2012-3552/draft-guidance-for-industry-on-scientific-considerations-in-demonstrating-biosimilarity-to-a
2015	FDA finalizes biosimilarity guidance; first U.S. biosimilar (Zarxio) approved; tiered analytical testing framework established	https://www.federalregister.gov/documents/2015/04/30/2015-10062/scientific-considerations-in-demonstrating-biosimilarity-to-a-reference-product-guidance-for; https://www.fda.gov/vaccines-blood-biologics/general-biologics-guidances/biosimilars-guidances
2016	FDA removes Analytical Similarity Testing guideline in response to author’s petition/	Citizen Petition FDA-2016-P-1871; https://www.regulations.gov
2017	FDA Biosimilars Action Plan launched to streamline approval process	https://www.fda.gov/drugs/biosimilars/biosimilars-action-plan; https://www.fda.gov/media/114574/download
2018	FDA acknowledges need for overhauling biosimilar regulatory guidance in response to author’s petition.	https://www.regulations.gov/document/FDA-2018-P-1876-0001
2019	FDA guidance acknowledges potential for CES waivers; first interchangeable biosimilar approved	Citizen Petition FDA-2019-P-1236; https://www.regulations.gov/document?D=FDA-2019-P-1236-0001
2022	FDA Modernization Act 2.0 eliminates the statutory mandate for animal testing; FDA removes tiered analytical testing; PD biomarker guidance published	FDA Modernization Act 2.0; https://www.congress.gov/bill/117th-congress/senate-bill/2952; Niazi SK. Science. 2022;377(6602):162–163
2023	FDA releases GASK guidance enabling reliance on established scientific knowledge; biosimilar labeling guidance updated to remove interchangeability mention	Citizen Petition FDA-2023-P-3766; https://www.regulations.gov/document/FDA-2023-P-3766-0001; https://www.fda.gov/regulatory-information/search-fda-guidance-documents/labeling-biosimilar-and-interchangeable-biosimilar-products
Jan 2025	EMA publishes reflection paper on tailored clinical approach in biosimilar development	EMA/CHMP/BMWP/60916/2025; https://www.ema.europa.eu/en/reflection-paper-tailored-clinical-approach-biosimilar-development
Apr 2025	Expedited Access to Biosimilars Act (S.1441) was introduced in the U. S. Senate	S.1441; https://www.congress.gov/bill/119th-congress/senate-bill/1441
Sep 2025	FDA issues final Analytical guide to establish biosimilarity that was removed in 2016	https://www.fda.gov/regulatory-information/search-fda-guidance-documents/development-therapeutic-protein-biosimilars-comparative-analytical-assessment-and-other-quality
Sep 2025	FDA accepts first-ever CES waiver for monoclonal antibody biosimilar application filed by the author.	https://www.fda.gov/regulatory-information/search-fda-guidance-documents/scientific-considerations-demonstrating-biosimilarity-reference-product-updated-recommendations
Oct 2025	The FDA issues draft guidance proposing streamlined requirements for the CES requirement for all biosimilars; streamlined nonclinical guidance for mAbs is released	Docket FDA-2011-D-0605; https://www.fda.gov/regulatory-information/search-fda-guidance-documents/scientific-considerations-demonstrating-biosimilarity-reference-product-updated-recommendations; https://www.fda.gov/news-events/press-announcements/fda-moves-accelerate-biosimilar-development-and-lower-drug-costs
Dec 2025	Comprehensive citizen petition filed by the author advocating additional reforms: USP specifications, automatic interchangeability, immunogenicity waivers	https://www.regulations.gov/document/FDA-2025-P-6763-0003 https://www.regulations.gov/docket/FDA-2024-P-3822

#### 3.1.1 From comparative efficacy requirements to analytical primacy

Early biosimilar guidance documents, including the (12) framework and the EMA’s initial guidelines, emphasized comparative clinical efficacy studies as central evidentiary requirements (32, 33). The prevailing regulatory logic assumed that clinical endpoints provided essential confirmation of therapeutic equivalence that could not be reliably inferred from analytical or pharmacokinetic data alone. However, accumulated regulatory experience and peer-reviewed analyses progressively challenged this assumption (34–36).

Retrospective analyses of approved biosimilars demonstrated that comparative clinical efficacy trials consistently confirmed the biosimilarity conclusions already established through analytical characterization, without providing independent decision-relevant information that altered approval outcomes (37). Published literature examining regulatory submissions concluded that no biosimilar approval decision had been reversed or substantially modified based on clinical efficacy data (38) when robust analytical similarity had been demonstrated (30, 39). These findings provided the empirical foundation for regulatory reconsideration of mandatory efficacy testing requirements.

Niazi’s comprehensive analysis of over 600 biosimilar studies documented that no biosimilar with proven analytical similarity has ever failed a comparative efficacy study (40), fundamentally questioning the scientific rationale for maintaining these costly requirements (41). This body of evidence demonstrated that comparative efficacy trials function as confirmatory rather than discriminatory tests, incapable of detecting differences not already apparent through comprehensive analytical assessment.

#### 3.1.2 Elimination of mandatory animal testing

A landmark transformation occurred with the passage of the FDA Modernization Act 2.0 in December 2022, which removed the statutory mandate for animal testing before human clinical trials (42). This legislative change reflected scientific consensus that animal studies for biosimilar monoclonal antibodies targeting well-characterized pathways provide limited translational value (43) beyond what is predicted by mechanism-of-action analysis and prior reference product experience (44, 45).

Published studies in peer-reviewed literature have documented that animal toxicology findings for biosimilar antibodies rarely yielded decision-relevant information (46) not already available from reference product labeling and mechanistic understanding. Niazi’s influential letter in *Science* magazine catalyzed this regulatory shift by presenting compelling evidence that animal testing for biosimilars is unnecessary and lacks commensurate scientific value (47).

The FDA’s 2025 draft guidance on streamlined nonclinical safety studies for monospecific monoclonal antibodies formalized this evolution, establishing that comprehensive weight-of-evidence approaches may replace animal testing (48) when prior knowledge from the reference product and therapeutic class sufficiently characterizes expected toxicities (49). This represents a fundamental departure from the assumption that animal studies provide essential safety assurance for all biologic development programs, and aligns with the FDA’s broader Roadmap to Reducing Animal Testing in Preclinical Safety Studies (50, 51).

#### 3.1.3 Codification of waiver pathways for comparative efficacy studies

The FDA’s October 2025 draft guidance explicitly addresses circumstances under which comparative clinical efficacy studies may be waived for biosimilar development (2). This guidance represents the culmination of evolving regulatory thinking documented in peer-reviewed literature and formal regulatory submissions advocating for evidence-based reform (41, 52). The guidance establishes that waiver determinations should be based on the residual uncertainty remaining after comprehensive analytical and pharmacokinetic evaluation, rather than on prescriptive requirements applied uniformly across all products.

In September 2025, the FDA accepted the first-ever CES waiver for a monoclonal antibody biosimilar application, marking a precedent-setting regulatory breakthrough. This decision validated the scientific arguments advanced through multiple citizen petitions and peer-reviewed publications demonstrating that analytical similarity and pharmacokinetic equivalence, when rigorously established, provide sufficient evidentiary foundation for biosimilarity conclusions without the need for redundant clinical efficacy trials (53, 39, 54).

The draft guidance addressing CES requirements released in October 2025 by FDA Commissioner Marty Makary was described as potentially reducing biosimilar development timelines by 3–4 years, fundamentally reshaping the economics of biosimilar development, and enabling smaller generic drug manufacturers to enter the field (55). Proposals for global harmonization of biosimilar guidelines have been advanced through the International Council for Harmonisation framework, reflecting recognition that divergent regional requirements create inefficiencies without enhancing patient safety (56).

#### 3.1.4 Recognition of generally accepted scientific knowledge

Regulatory frameworks have increasingly recognized the role of generally accepted scientific knowledge (GASK) in supporting biosimilar development decisions. The FDA’s May 2023 guidance on GASK provided a formal mechanism for developers to leverage accumulated scientific understanding of monoclonal antibody structure–function relationships, manufacturing platform consistency, and therapeutic class behavior to reduce redundant empirical testing requirements (57).

The integration of GASK principles into regulatory decision-making represents a maturation of the biosimilar framework toward evidence-efficient development paradigms. Published analyses have identified the opportunity to apply GASK to support waivers of pharmacodynamic testing, clinical efficacy studies, and specific immunogenicity assessments when the totality of scientific evidence supports predictable outcomes (58, 59).

#### 3.1.5 Advancement of analytical similarity assessment

Analytical similarity assessment has become the dominant evidentiary pillar of biosimilar antibody development, reflecting both technological advancement and accumulated regulatory experience demonstrating the predictive power of comprehensive molecular characterization. Advances in mass spectrometry (including peptide mapping, intact mass analysis, and hydrogen-deuterium exchange), higher-order structure characterization (circular dichroism, Fourier-transform infrared spectroscopy, differential scanning calorimetry), and functional bioassays (60) now allow molecular resolution that far exceeds what was available during the early biosimilar era (34, 61, 62).

Orthogonal analytical methods can define a multidimensional similarity ‘fingerprint’ encompassing primary structure identity, conformational integrity, glycosylation patterns, charge variant distribution, aggregation propensity, and functional activity across multiple mechanisms of action. The development of AI-driven paradigms for molecular biosimilarity assessment, including the application of protein structure prediction algorithms (63), has further enhanced the precision of analytical comparisons (64).

Proposals to enable the U.S. Pharmacopeia to create Biological Product Specifications (BPS) would further streamline analytical assessment by establishing standardized product release specifications and validated testing methods, removing the need for developers to procure multiple reference product lots for side-by-side comparison (65). Regulatory agencies consistently emphasize that the extent of residual uncertainty remaining after comprehensive analytical comparison determines the need for additional nonclinical or clinical data (32, 33, 66, 67).

#### 3.1.6 Future directions and remaining challenges

The collective trajectory of these regulatory transformations demonstrates a systematic shift from assumption-based testing requirements toward science-driven, risk-proportionate frameworks. This evolution reflects both technological advancement in analytical characterization and the accumulated empirical record demonstrating the predictive reliability of comprehensive molecular assessment for monoclonal antibody biosimilars.

Additional reforms advocated in recent citizen petitions include: automatically designating all FDA-approved biosimilars as interchangeable, replacing routine immunogenicity studies with validated aggregate-removal technologies, standardizing analytical testing through USP Biological Product Specifications, and harmonizing with international regulatory frameworks (52). Legislative initiatives such as the Expedited Access to Biosimilars Act (S.1441) would codify many of these reforms at the statutory level.

The implementation of continuous manufacturing technologies for recombinant proteins offers additional opportunities to reduce biosimilar production costs and enhance product quality consistency (68). Current projections suggest that biosimilars enabled by streamlined regulatory pathways and continuous manufacturing technologies could generate savings of $125–237 billion in the United States alone between 2023 and 2027, with individual patients potentially saving $1,800–5,500 annually through increased access to cost-effective biological therapeutics.

The evolution of FDA biosimilar regulations from 2010 to 2025 represents one of the most significant transformations in pharmaceutical regulatory science. The systematic elimination of mandatory animal testing, the removal of blanket comparative efficacy study requirements, recognition of GASK principles, and the advancement of analytical similarity assessment have collectively created a regulatory pathway that balances evidence efficiency with robust safety and efficacy standards.

These changes, driven primarily by persistent scientific advocacy and the generation of empirical evidence, have fundamentally reshaped the biosimilar development landscape. The resulting framework enables development costs approaching those of abbreviated new drug applications, facilitates market access for over 100 biological molecules currently without biosimilar competition, and positions biosimilars to fulfill their original promise of bringing affordable biological medicines to patients worldwide.

### 3.2 New guidance needed

The proposed draft guidance articulates principles that directly support NSPT classification, particularly the principle that comparative clinical efficacy studies should be reserved to address specific residual uncertainties that cannot be resolved through analytical and functional characterization. For products whose mechanism of action is well understood, and functional assays directly measure clinically relevant activity, the guidance suggests that robust analytical similarity, combined with comparable pharmacokinetics, may suffice for approval. This risk-based approach to evidence generation mirrors the scientific rationale underlying NSPT classification, where products with bounded, measurable pharmacology require less extensive clinical confirmation than those with complex, amplifying mechanisms.

The FDA’s Purple Book database provides the operational infrastructure for implementing NSPT classification within the existing regulatory framework (3). This authoritative repository of licensed biological products and their biosimilar relationships enables systematic identification of NSPT-eligible reference products and tracking of biosimilar approvals. Analysis of Purple Book data through November 2025 reveals forty-three currently licensed biological products that meet NSPT criteria based on their mechanisms of action, providing a substantial initial cohort for classification. The database structure, with its clear delineation between reference products approved under section 351(a) and biosimilars approved under section 351(k) of the Public Health Service Act, facilitates assessment of competitive dynamics and identification of biosimilar void opportunities.

Historical regulatory precedents demonstrate the FDA’s capacity to adapt evidence requirements based on mechanisms. The March 2020 transition of certain biological products from regulation under the Federal Food, Drug, and Cosmetic Act to the Public Health Service Act exemplifies regulatory modernization in response to scientific understanding and competitive dynamics (12, 13). Insulin products, previously approved as new drug applications despite their biological nature, were transitioned to biologics license applications to enable biosimilar competition under the 351(k) pathway. While insulin itself functions as a receptor agonist outside the NSPT framework, the transition demonstrates regulatory flexibility in reclassifying products to align approval pathways with scientific characteristics and competitive objectives.

The approval of somatropin products under abbreviated pathways before the establishment of the formal biosimilar framework constitutes another instructive precedent. Omnitrope received FDA approval in 2006 under section 505(b)(2) of the Federal Food, Drug, and Cosmetic Act, demonstrating that abbreviated development based on analytical and functional similarity was scientifically justified even before the Biologics Price Competition and Innovation Act created the 351(k) biosimilar pathway (69). This precedent supports the principle that mechanism-based evidence requirements can be implemented within existing regulatory structures when scientific justification exists.

International regulatory divergence in the classification of biological products creates both challenges and opportunities for NSPT implementation. The European Medicines Agency has consistently classified certain products as biosimilars, even though they are regulated differently in the United States, most notably insulin analogs and somatropin products. Insulin glargine biosimilar Abasaglar received EMA approval explicitly as a biosimilar to the reference product Lantus. In contrast, in the United States, similar products initially followed different regulatory pathways before the 2020 transition (70). This divergence highlights the need for a harmonized, mechanism-based classification that could facilitate global development programs and reduce the generation of duplicative evidence across jurisdictions.

The regulatory environment also increasingly recognizes the limitations of comparative clinical efficacy studies for detecting meaningful differences between biosimilars and reference products. These studies typically require thousands of patients and extended treatment periods to achieve adequate statistical power to demonstrate equivalence, yet rarely identify clinically relevant differences even when analytical and functional similarity has been established. The low discriminatory power of clinical endpoints for detecting differences that matter for product performance, combined with the high cost and long timelines of clinical trials, creates strong scientific and economic arguments for mechanism-based evidence requirements that reserve clinical studies for situations where they add meaningful information.

Recent FDA guidance on analytical similarity assessment further supports the technical feasibility of NSPT classification by establishing sophisticated frameworks for evaluating critical quality attributes and their relationships to clinical performance (71). Advanced analytical techniques, including mass spectrometry, nuclear magnetic resonance, and biophysical characterization methods, can now resolve structural differences at the atomic level. At the same time, cell-based potency assays provide sensitive measures of functional activity. For NSPTs in which therapeutic effect correlates directly with measurable biochemical activity, these analytical and functional assessments offer more sensitive and mechanistically relevant evidence than clinical trials powered for non-inferiority.

The evolving regulatory landscape also reflects growing recognition that biosimilar development should focus on detecting clinically relevant differences rather than documenting similarity through redundant evidence. The totality-of-evidence approach has always emphasized using the most sensitive methods to detect meaningful differences, but historical practice often defaulted to clinical studies regardless of their discriminatory power. The NSPT framework operationalizes this principle by identifying products in which analytical and functional methods are inherently more sensitive than clinical endpoints for detecting differences that could affect therapeutic performance.

## 4 Mechanistic rationale: why NSPTs exhibit predictable pharmacology

The scientific foundation for streamlined NSPT biosimilar development rests on fundamental differences in how these products exert therapeutic effects compared to receptor-signaling biologics. Understanding these mechanistic distinctions explains why functional assays provide reliable surrogates for clinical performance in NSPTs yet remain insufficient for products that trigger complex signaling cascades. The predictability of NSPT pharmacology arises from three interconnected principles: a bounded biological response, a direct correlation between biochemical activity and clinical effect, and the absence of signal amplification through intracellular cascades.

The concept of a bounded biological response distinguishes NSPTs from receptor-signaling therapeutics with respect to their dose–response relationships and maximal achievable effects. For ligand-neutralizing antibodies, the maximum therapeutic effect occurs when sufficient drug is present to neutralize all available target ligand, creating a ceiling effect that depends primarily on drug exposure and target production rates rather than complex downstream biology. Anti-TNF therapeutics exemplify this principle, in which clinical anti-inflammatory effects plateau once TNF-alpha is sufficiently neutralized, regardless of further dose escalation (20). This bounded response contrasts sharply with receptor agonists, in which increasing receptor occupancy can trigger exponentially amplifying responses through signal cascade activation, with effects that vary dramatically depending on tissue-specific receptor expression and the availability of downstream signaling components.

Direct measurement of clinically relevant activity via functional assays is a critical advantage in NSPT biosimilar development. For these products, the biochemical activity measured in validated potency assays directly reflects the mechanism driving therapeutic benefit. Cell-based neutralization assays for anti-cytokine antibodies assess the specific mechanism by which antibodies prevent receptor activation in patients. Hemolysis inhibition assays for complement blockers quantify the same cascade interruption that prevents complement-mediated cell destruction *in vivo* (24). This direct relationship between measurable potency and clinical effect enables functional similarity to serve as a reliable predictor of therapeutic equivalence.

Signal transduction cascades introduce multiple layers of biological amplification and context-dependence, complicating biosimilar assessment for receptor-engaging therapeutics. When a growth factor or agonistic antibody binds its receptor, the initial binding event triggers conformational changes that activate intracellular kinases, which phosphorylate multiple substrates, thereby activating transcription factors and altering gene expression, ultimately determining the therapeutic response. Each step in this cascade can amplify or attenuate the initial signal, depending on factors such as cofactor availability, phosphatase activity, feedback-inhibitor expression, and crosstalk with other signaling pathways. Minor differences in binding kinetics or receptor clustering induced by structural variants can propagate through these cascades, resulting in meaningful differences in biological responses.

NSPTs bypass this complexity by acting upstream of receptor engagement, intercepting ligands or blocking cascades before signal amplification occurs. The pharmacology remains linear and predictable because the therapeutic effect depends primarily on reducing effective concentrations of target molecules below thresholds required for biological activity. For anti-VEGF therapeutics, clinical benefit in diabetic macular edema and age-related macular degeneration correlates directly with the degree of VEGF suppression achievable in ocular compartments, a parameter measurable through validated neutralization assays (72, 73). Similarly, for anti-interleukin therapeutics targeting IL-17A, IL-23, IL-5, and IL-12/23, therapeutic effects in psoriasis, asthma, and inflammatory bowel disease correlate with the degree of cytokine neutralization rather than with complex downstream immunological reprogramming.

The absence of tissue-specific contextual effects further supports the predictability of NSPT pharmacology. Receptor-signaling biologics often exhibit variable effects across tissues based on differential receptor expression, cofactor availability, and local signaling environments. A receptor agonist might trigger proliferation in one tissue while inducing differentiation or apoptosis in another based on the constellation of downstream signaling components present. NSPTs avoid this complexity because their mechanism, ligand sequestration or cascade blockade, operates identically across tissue contexts. TNF-alpha neutralization prevents inflammatory signaling equally in synovial joints, intestinal mucosa, and skin, differing only in local drug exposure rather than fundamental mechanism.

Pharmacokinetic-pharmacodynamic relationships for NSPTs typically follow predictable patterns amenable to modeling and simulation (74). Straightforward equilibrium binding equations can describe the relationship between drug concentration and target suppression, provided they are modified to account for *in vivo* ligand production and clearance rates. For products such as denosumab that neutralize RANKL, the degree of suppression of bone resorption markers correlates directly with circulating drug levels in a predictable manner across osteoporosis and skeletal-related event-prevention indications. This predictability, combined with pharmacokinetic similarity and comparable potency, provides strong evidence for biosimilar therapeutic equivalence without the need for extensive clinical confirmation.

The concept of functional redundancy in NSPT mechanisms provides additional scientific support for streamlined biosimilar development. Many NSPTs target ligands or cascade components, where complete suppression is neither necessary nor achieved for therapeutic benefit. Partial neutralization of inflammatory cytokines or incomplete complement blockade can yield substantial clinical improvement, creating a therapeutic window in which minor differences in potency or pharmacokinetics have limited clinical impact. This functional redundancy contrasts with receptor-signaling systems, where small changes in activation can tip the balance between proliferation and apoptosis or between immune activation and tolerance.

## 5 Proposed evidence package for NSPT biosimilars

The evidence requirements for NSPT biosimilars should reflect the predictable relationships among analytical attributes, functional activity, and clinical performance characteristics of these products, while acknowledging the ethical imperative to eliminate unnecessary studies that delay patient access without adding scientific value. We propose a fit-for-purpose evidence package that prioritizes mechanistically relevant assessments while reserving clinical studies for addressing specific residual uncertainties. This framework aligns with FDA modernization principles and provides clear expectations that facilitate efficient development planning and regulatory review. Table 2 presents a comprehensive evidence framework to guide NSPT biosimilar development, emphasizing analytical and functional characterization as the primary determinants of biosimilarity.

**Table 2:** Proposed evidence framework for NSPT biosimilar development.

Evidence component	Primary assessment	Acceptance criteria	Contingency triggers	References supporting approach
Structural characterization	Primary sequence confirmation via mass spectrometry and peptide mapping	100% sequence identity, including post-translational modifications	Any sequence variation requires justification	(71)
Higher-order structure	Multi-method assessment including circular dichroism, FTIR, NMR, and HDX-MS	Statistical similarity within the variability of reference product batches	Differences in critical structural regions trigger enhanced functional assessment	(71)
Glycosylation analysis	Site-specific glycan profiling via mass spectrometry; functional impact assessment for Fc glycans	Glycan profiles within the range of reference lots; no novel glycoforms impacting function	Differences affecting FcγR binding or complement activation require functional bridging	(78)
Charge variant profile	Ion exchange chromatography, capillary isoelectric focusing, and imaged capillary isoelectric focusing	Central peak and variant distribution within reference product range	Basic or acidic variants outside the reference range require isolation and characterization	(71)
Size variants	SEC-MALS, analytical ultracentrifugation, dynamic light scattering	Monomer content ≥ reference product; aggregates and fragments within specifications	Elevated aggregates trigger enhanced immunogenicity assessment	(71)
Process-related impurities	Host cell proteins, DNA, leachables, process additives	Below established safety thresholds; comparable to or lower than the reference product	Novel impurities require toxicological assessment	(32)
Functional characterization	Mechanism-specific potency assays, binding kinetics, and effector functions where relevant	Potency within 80–125% of reference; comparable dose–response curves	Potency differences > 10% trigger PK/PD assessment	(23)
Stability and degradation	Real-time, accelerated, and stress stability; forced degradation comparability	Comparable degradation pathways and rates; similar stability-indicating profiles	Novel degradation products require characterization and safety assessment	(71)
Pharmacokinetics	Comparative PK in the most sensitive population	90% CI for AUC and Cmax ratios within 80–125%	PK differences trigger exposure-response evaluation	(2)
Immunogenicity	Risk-based assessment; analytical prediction tools; clinical assessment, if warranted	No increased immunogenicity risk based on analytical assessment	Structural differences predicting immunogenicity trigger clinical evaluation	(2)

The analytical similarity assessment underpins the NSPT biosimilar demonstration, with an emphasis on orthogonal methods that probe different aspects of structure and function. Confirmation of the primary structure through multiple mass spectrometric approaches ensures complete sequence identity, including disulfide bond patterns and post-translational modifications. Higher-order structure assessment using biophysical methods sensitive to secondary, tertiary, and quaternary structure provides confidence that the three-dimensional architecture responsible for target binding is preserved. For products in which Fc-mediated effector functions contribute to the mechanism (82), detailed glycosylation analysis ensures comparable antibody-dependent cellular cytotoxicity (83), wherever relevant, complement-dependent cytotoxicity.

Functional characterization for NSPTs should emphasize mechanism-specific potency assays that directly measure the therapeutic activity. For ligand-neutralizing antibodies, this includes determining binding affinity by surface plasmon resonance or bio-layer interferometry and performing cell-based neutralization assays using relevant target cell lines. Multiple orthogonal functional assays provide convergent evidence of similarity while identifying subtle differences that require further investigation. For anti-TNF biosimilars, a panel comprising TNF-binding kinetics, neutralization of TNF-induced cytotoxicity, inhibition of TNF-triggered cytokine release, and blockade of TNF-mediated cell activation provides a comprehensive functional assessment (23).

Comparative pharmacokinetic assessment remains essential for NSPT biosimilars despite the streamlined clinical package, as drug exposure directly determines the extent of target neutralization or cascade blockade achievable *in vivo* (84). The pharmacokinetic study should be conducted in the most sensitive population to detect potential differences, typically healthy volunteers for products without safety concerns, or patients with the approved indication when therapeutic proteins are not suitable for healthy volunteer studies. The crossover or parallel group design should provide adequate statistical power to demonstrate pharmacokinetic similarity within conventional bioequivalence margins. For products with nonlinear pharmacokinetics due to target-mediated drug disposition, studies should span the dose range where sensitivity to detect differences is greatest.

The immunogenicity assessment strategy for NSPTs should be risk-based (85), leveraging analytical tools to predict immunogenic potential while reserving clinical immunogenicity studies for situations where analytical assessment identifies concerning differences. In silico and *in vitro* methods, including T-cell epitope prediction, dendritic cell activation assays, and cytokine release assessments, can identify structural features likely to trigger immune responses (86). For most NSPTs where analytical similarity is demonstrated and no novel immunogenic features are identified, post-marketing immunogenicity surveillance may suffice rather than powered pre-approval clinical studies. This approach recognizes that immunogenicity for well-characterized proteins is primarily driven by product-related factors detectable by analytical methods (87), thereby obviating the need for large clinical trials for assessment.

Clinical studies should be triggered only when specific residual uncertainties remain after comprehensive analytical and functional characterization. These triggers might include analytical differences in critical quality attributes that could affect clinical performance, functional differences exceeding pre-specified similarity margins in mechanism-relevant assays, pharmacokinetic differences suggesting altered exposure-response relationships, or immunogenicity signals from analytical assessment that require clinical confirmation. The clinical study design should be tailored to address the specific uncertainty rather than defaulting to large equivalence trials. For example, if the only residual uncertainty concerns immunogenicity, a focused immunogenicity study with an appropriate sample size and duration would suffice rather than a full comparative efficacy trial.

The evidence package should also include, when available, pharmacodynamic markers directly related to the mechanism of action. For anti-RANKL antibody biosimilars, bone turnover markers, including C-terminal telopeptide and N-terminal propeptide of type I collagen, provide direct evidence of biological activity equivalence. For complement inhibitors, markers such as CH50, alternative pathway hemolytic activity, and soluble C5b-9 levels indicate comparable blockade of the complement cascade. These pharmacodynamic assessments bridge between functional assays and clinical outcomes, providing additional confidence in therapeutic equivalence without requiring extensive clinical endpoint studies.

**Figure 1:**
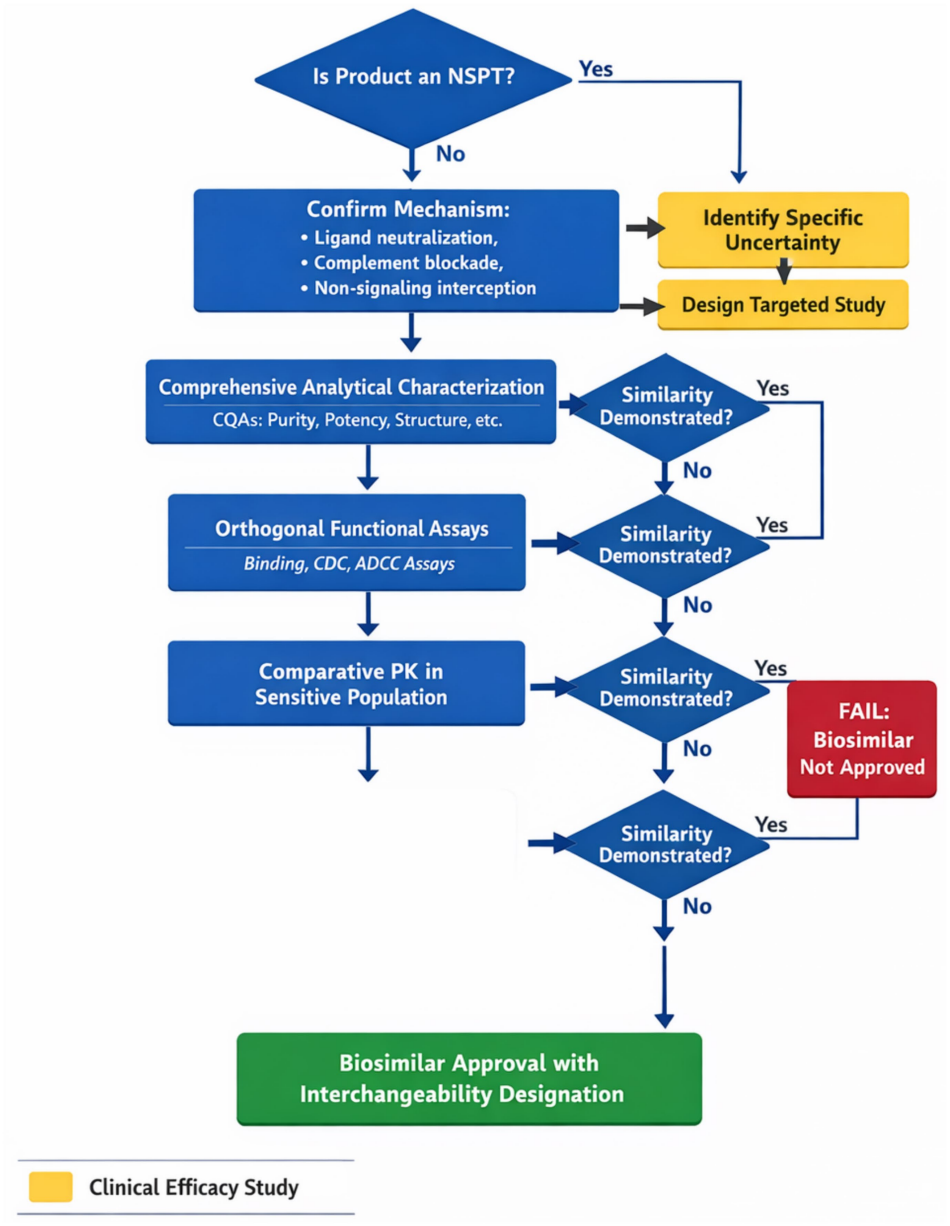
NSPT decision making tree.

Figure 1 illustrates the decision framework for NSPT biosimilar development, demonstrating how the pathway prioritizes analytical and functional evidence while reserving clinical studies for specific uncertainties. This framework operationalizes the ethical principle that every clinical trial should address a genuine scientific question rather than serve as a regulatory checkbox.

The proposed evidence framework explicitly acknowledges that different NSPTs may require tailored approaches based on their specific mechanisms and therapeutic applications. Anti-VEGF agents used in ophthalmology may require specialized assessments of aggregation and particulate matter given intravitreal administration, while systemically administered anti-cytokine antibodies may focus more on systemic exposure and immunogenicity. The framework provides sufficient flexibility to accommodate these product-specific considerations while maintaining the core principle that clinical efficacy studies should address specific uncertainties rather than serve as routine requirements.

## 6 Ethical imperatives: the human cost of regulatory inefficiency

The economic rationale for NSPT classification complements broader considerations regarding resource allocation and patient access. The current regulatory framework that applies extensive clinical trial requirements uniformly to biosimilars, regardless of mechanism-based predictability, warrants examination from a resource stewardship perspective. Understanding the implications of current evidence requirements provides context for evaluating whether NSPT classification guidelines merit consideration by the FDA and EMA.

Clinical trials that confirm findings already established through analytical characterization represent a potential opportunity cost in research resource allocation. A typical comparative efficacy study for an NSPT biosimilar enrolls 500–1,000 patients, costs 50–100 million dollars (estimated), and requires 2–3 years to complete—resources that could alternatively support early-phase studies of novel therapeutics or expanded access programs. When such trials consistently confirm analytical similarity findings without providing additional decision-relevant information, their incremental scientific value warrants careful consideration against resource allocation principles.

The biosimilar void documented by IQVIA represents an area warranting analysis—products with expired patents and established mechanisms that have not attracted biosimilar development (6). Table 3 presents NSPT products where limited biosimilar competition may be associated with regulatory uncertainty regarding evidence requirements. Patient estimates in Table 3 are derived from epidemiological literature and may vary based on disease prevalence assumptions and treatment eligibility criteria; economic projections represent potential rather than guaranteed outcomes (Figure 1).

**Table 3:** The biosimilar void: NSPT products with limited competition despite patent expiration.

Reference product	Mechanism	Patient population	Annual cost per patient	Years since patent expiry	Estimated patients denied access	Unnecessary trial burden
Secukinumab (Cosentyx)	IL-17A neutralization	Psoriasis, psoriatic arthritis	$65,000–85,000	2	>50,000 annually	3 Phase III trials, 2,400 patients
Guselkumab (Tremfya)	IL-23p19 neutralization	Plaque psoriasis	$70,000-90,000	Approaching	>30,000 annually	2–3 trials, 1,800 patients
Evolocumab (Repatha)	PCSK9 neutralization	Hypercholesterolemia	$6,000-8,000	3	>200,000 annually	CV outcome trial concerns
Alirocumab (Praluent)	PCSK9 neutralization	Hypercholesterolemia	$6,000-8,000	3	>150,000 annually	CV outcome trial concerns
Mepolizumab (Nucala)	IL-5 neutralization	Severe eosinophilic asthma	$35,000-40,000	Approaching	>15,000 annually	2 trials, 800 patients
Omalizumab (Xolair)	IgE neutralization	Allergic asthma	$30,000-40,000	5	>100,000 annually	Multiple indication trials
Canakinumab (Ilaris)	IL-1β neutralization	Rare inflammatory diseases	$200,000–400,000	4	>2,000 annually	Orphan population trials

Sources: IQVIA Institute (4); pricing from published formulary data; patient estimates from epidemiological studies.

The human impact extends beyond simple access denial. Patients forced to ration or discontinue biological therapies due to cost experience disease progression, reduced quality of life, and preventable morbidity. Healthcare systems divert resources from other priorities to cover monopolistic pricing maintained by regulatory barriers rather than genuine innovation. Clinicians experience moral distress when optimal therapies remain financially inaccessible to their patients due to the absence of biosimilar competition, which could reduce costs by 30–70% based on established market dynamics (8).

The concentration of biosimilar development among first-generation NSPTs, despite the absence of competition among newer products with identical mechanisms, indicates that uncertainty about regulatory requirements, not scientific complexity, drives the biosimilar void. Sponsors rationally avoid investing in development programs where clinical trial expectations remain opaque and potentially prohibitive. This regulatory uncertainty effectively extends monopolies beyond patent protection (88), transferring billions from healthcare systems to originator companies without corresponding innovation benefits—a wealth transfer that violates principles of distributive justice and efficient resource allocation.

Geographic disparities in biosimilar availability further illuminate the ethical dimensions of regulatory inefficiency. The European Medicines Agency has approved biosimilars for golimumab and other NSPTs not available in the US, demonstrating that scientific evidence supports streamlined development while regulatory divergence maintains artificial barriers (89). Patients in countries with clearer regulatory frameworks gain earlier access to affordable biosimilars (90). At the same time, those in jurisdictions with ambiguous requirements suffer prolonged exposure to monopolistic pricing—an arbitrary inequality based on geography rather than medical need.

The PCSK9 inhibitor category exemplifies the human cost of regulatory uncertainty for NSPTs with precise mechanisms and substantial public health impact. Evolocumab and alirocumab, together, serve patients with hypercholesterolemia through well-characterized PCSK9 neutralization that prevents LDL receptor degradation—a mechanism directly measurable in functional assays (91, 92). Despite straightforward neutralization pharmacology and expired patents, no biosimilars have entered development due to uncertainty about cardiovascular outcome trial requirements, which could cost hundreds of millions and take 5–7 years to complete. This regulatory paralysis denies hundreds of thousands of patients access to cholesterol management that could prevent cardiovascular events and save lives.

**Figure 2:**
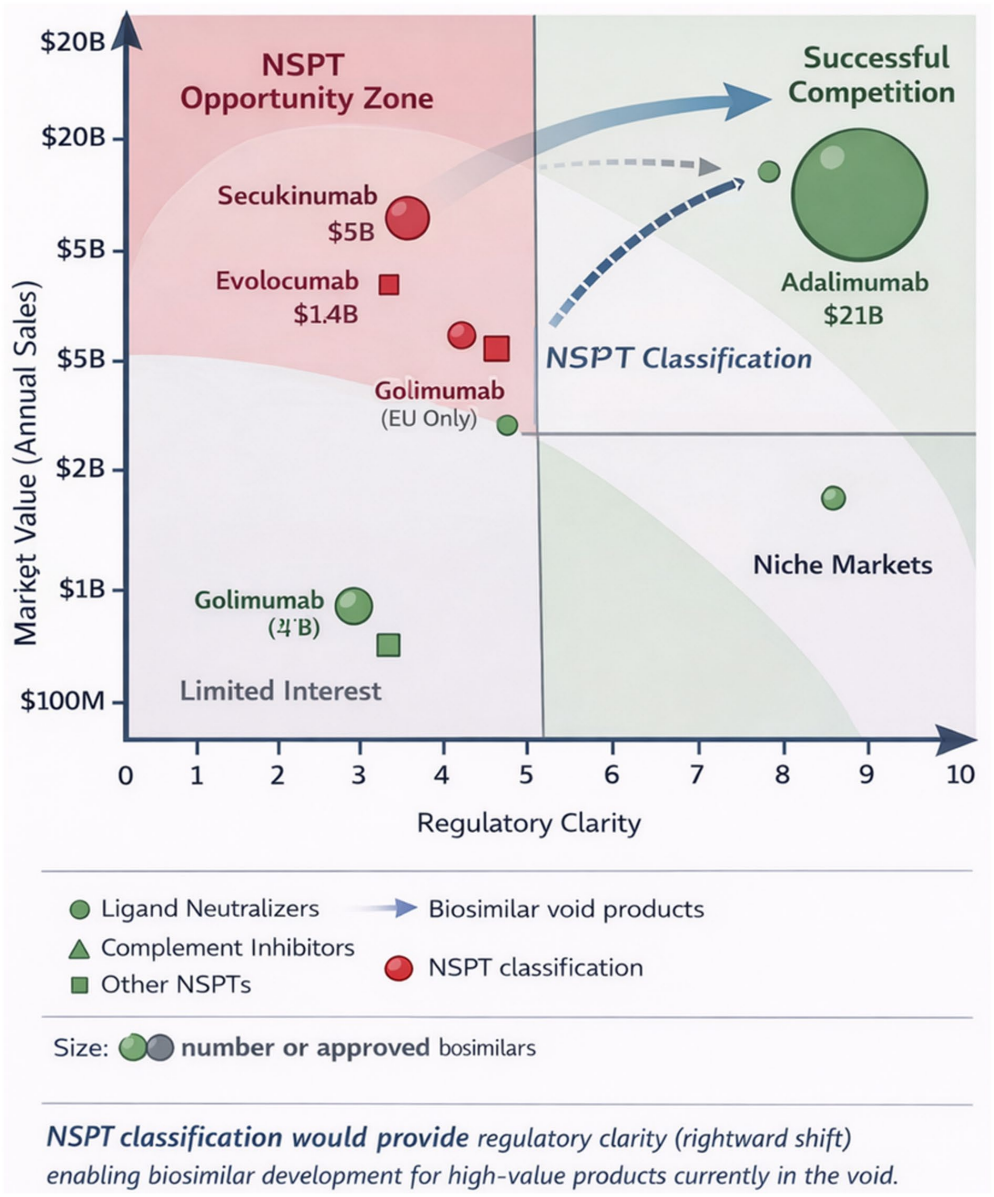
Case study evidence for NSPT classification. This Figure presents a conceptual synthesis derived from published market and regulatory analyses; it does not represent primary data collection.

Figure 2 illustrates the relationship among mechanism complexity, regulatory uncertainty, and the resulting biosimilar void, demonstrating how NSPT classification could enable competition for products currently trapped in regulatory limbo.

The scientific validity of NSPT classification is best demonstrated through a detailed examination of specific products in which mechanism-based evidence packages have been successfully implemented or in which accumulated data support streamlined development approaches. These case studies span multiple therapeutic areas and mechanisms, providing concrete evidence that functional characterization can reliably predict clinical performance for non-signaling therapeutics.

### 6.1 Anti-TNF therapeutics: archetypal NSPTs with proven biosimilar success

The anti-tumor necrosis factor alpha therapeutic class provides the most comprehensive evidence supporting NSPT classification, with extensive biosimilar development experience demonstrating that analytical and functional similarity reliably predict clinical equivalence. Five anti-TNF products have been developed as originator biologics, including three monoclonal antibodies (adalimumab, infliximab, golimumab), one pegylated Fab fragment (certolizumab pegol), and one receptor fusion protein (etanercept), all sharing the common mechanism of neutralizing soluble and membrane-bound TNF-alpha to prevent receptor activation (20).

The clinical effects of anti-TNF therapeutics correlate directly with the degree of TNF neutralization at sites of inflammation, a relationship validated across multiple indications, including rheumatoid arthritis, psoriatic arthritis, ankylosing spondylitis, inflammatory bowel disease, and psoriasis. Pharmacokinetic-pharmacodynamic modeling demonstrates that clinical response plateaus once TNF is sufficiently neutralized, with further dose increases providing no additional benefit. This bounded response profile contrasts sharply with receptor agonists, where dose escalation can trigger exponentially increasing biological effects through signal amplification.

Biosimilar development for anti-TNF products has validated the predictive value of functional assays for clinical performance. The FDA has approved multiple biosimilars for adalimumab, infliximab, and etanercept, based on evidence packages in which analytical and functional similarity, combined with comparable pharmacokinetics, provided the primary evidence of biosimilarity. Clinical efficacy studies, when conducted, consistently confirmed equivalence and identified no meaningful differences, demonstrating limited discriminatory power compared with analytical and functional assessments. The European experience with golimumab biosimilars, in which products were approved based on comprehensive analytical packages with limited clinical evidence, further supports the adequacy of mechanism-based evidence.

Standardized functional assay panels for anti-TNF biosimilars have emerged through regulatory experience, providing clear benchmarks for similarity assessment. These panels typically include TNF-binding affinity measurements by surface plasmon resonance, neutralization of TNF-induced cytotoxicity in sensitive cell lines, inhibition of TNF-triggered inflammatory cytokine production, blockade of TNF-mediated endothelial cell activation, and prevention of TNF-induced apoptosis in relevant cell types. Convergent evidence from multiple orthogonal assays that measure the underlying neutralization mechanism provides robust confirmation of functional similarity, which directly translates into clinical equivalence.

### 6.2 Ophthalmologic NSPTs: aflibercept and ranibizumab

The anti-vascular endothelial growth factor therapeutics used in ophthalmology demonstrate how NSPT principles apply to specialized routes of administration and compartmentalized pharmacology. Aflibercept, a fusion protein that functions as a decoy receptor for VEGF-A, VEGF-B, and placental growth factor, exemplifies pure ligand-trap pharmacology, in which the therapeutic effect depends entirely on sequestering growth factors before they can engage their cognate receptors (73). Ranibizumab, a humanized antibody Fab fragment specific for VEGF-A, similarly functions by simple neutralization, without any signaling activity (22).

The ophthalmologic applications of these agents in age-related macular degeneration, diabetic macular edema, and retinal vein occlusion provide unique insights into NSPT pharmacology, as therapeutic effects occur in an anatomically isolated compartment where drug concentration and target neutralization can be directly correlated with clinical outcomes. Optical coherence tomography provides an objective, quantitative assessment of retinal thickness and fluid accumulation that directly reflects the degree of VEGF suppression achieved. This tight correlation between measurable pharmacodynamic effect and clinical benefit supports the use of functional assays as primary evidence for biosimilar approval.

Recent FDA approvals of aflibercept and ranibizumab biosimilars have relied heavily on analytical and functional similarity with streamlined clinical packages. The agency accepted a comprehensive analytical characterization demonstrating comparable protein structure, VEGF-binding affinity, neutralization potency, and pharmacokinetics following intravitreal injection. The limited clinical studies focused on confirming comparable efficacy using sensitive imaging endpoints rather than conducting large visual acuity trials. This regulatory precedent directly supports the NSPT framework, where mechanism-based functional evidence provides the primary basis for biosimilarity determination.

### 6.3 Denosumab: RANKL neutralization across multiple indications

Denosumab provides an instructive case study for NSPT classification because it demonstrates how a single neutralization mechanism translates into therapeutic benefit across diverse indications, ranging from osteoporosis to the prevention of skeletal-related events in cancer patients. The antibody functions exclusively through binding and neutralizing receptor activator of nuclear factor kappa-B ligand, preventing its interaction with RANK receptor on osteoclast precursors and thereby inhibiting bone resorption. This mechanism operates identically across postmenopausal osteoporosis, glucocorticoid-induced osteoporosis, and bone metastases, differing only in the degree of RANKL suppression required for therapeutic benefit in each condition.

The pharmacology of denosumab exhibits classic NSPT characteristics with predictable dose–response relationships and bounded therapeutic effects. Bone turnover markers, including serum C-terminal telopeptide and urinary N-terminal telopeptide, show rapid, dose-dependent suppression following denosumab administration, with maximal effects achieved at doses that fully neutralize RANKL. These biochemical markers directly reflect the degree of osteoclast inhibition and strongly correlate with long-term outcomes, including changes in bone mineral density and reductions in fracture risk. The ability to measure therapeutic mechanisms through validated biomarkers eliminates the need for long-term clinical endpoint studies in biosimilar development.

The first denosumab biosimilar approvals in 2024 validated the NSPT approach, with regulatory agencies accepting evidence packages emphasizing analytical and functional similarity. Critical assessments included RANKL binding affinity, neutralization of RANKL-induced osteoclastogenesis in cell culture systems, and inhibition of bone resorption in validated *in vitro* models. These functional assays directly measure the therapeutic mechanism, providing more sensitive and relevant evidence than clinical fracture studies that would require thousands of patients and years of follow-up to detect differences. The successful development of denosumab biosimilars, supported by mechanism-focused evidence packages, provides a template for future NSPT biosimilar programs.

### 6.4 Complement inhibitors: eculizumab and ravulizumab

The complement component C5 inhibitors eculizumab and ravulizumab exemplify NSPTs that function by interrupting the cascade rather than by neutralizing ligands, demonstrating the broader applicability of the non-signaling classification beyond cytokine-targeting antibodies. These therapeutics bind C5 to prevent its proteolytic cleavage into C5a and C5b, thereby blocking formation of the membrane attack complex without triggering any cellular signaling pathways (24, 25). The therapeutic effect in paroxysmal nocturnal hemoglobinuria, atypical hemolytic uremic syndrome, and myasthenia gravis depends entirely on the degree of complement blockade achievable at clinically relevant drug concentrations.

Functional assessment of complement inhibitors benefits from exceptionally well-validated assays that directly measure the therapeutic mechanism. The classical sheep erythrocyte hemolysis assay provides a quantitative measure of complement pathway blockade that directly correlates with clinical protection against complement-mediated cell lysis. Additional assays measuring CH50, alternative pathway activity, and soluble C5b-9 formation provide convergent evidence of complement inhibition. These functional assessments are far more sensitive than clinical endpoint studies for detecting differences in complement blockade, as demonstrated by their ability to distinguish between eculizumab and rovelizumab based on differences in C5 binding kinetics and duration of complement suppression, despite similar clinical efficacy.

The development of eculizumab biosimilars has progressed with regulatory acceptance of streamlined clinical packages that emphasize functional characterization. The high cost of these therapies, exceeding $500,000 annually for some indications, creates strong economic incentives for biosimilar development when regulatory requirements can be predicted with confidence. The NSPT framework would provide this clarity by establishing that complement inhibitors, as cascade-interrupting proteins with directly measurable functional effects, qualify for evidence packages emphasizing analytical and functional similarity rather than extensive clinical trials.

## 7 Global harmonization and the imperative for interchangeability

The international regulatory landscape for biological products reveals substantial divergence in classification and evidence requirements, complicating global biosimilar development and perpetuating access inequities based on geographic rather than scientific considerations. These differences are particularly pronounced for products that historically occupied regulatory gray zones between small molecules and biologics, resulting in cases in which identical products followed fundamentally different approval pathways across jurisdictions. The NSPT framework offers a scientifically grounded approach for harmonization that would benefit from coordinated regulatory action by the FDA and EMA to establish unified standards and automatic interchangeability designation for qualifying biosimilars.

The most instructive example of regulatory evolution concerns insulin products, which were regulated as drugs under the Federal Food, Drug, and Cosmetic Act in the United States. In contrast, they were treated as biological products eligible for biosimilar development in the European Union. Before March 2020, insulin products in the United States were approved through new drug application pathways, with follow-on products using the 505(b)(2) abbreviated pathway that required demonstration of pharmaceutical equivalence and bioequivalence rather than biosimilarity (12). Meanwhile, the European Medicines Agency explicitly classified insulin biosimilars within its biological product framework, approving products such as the insulin glargine biosimilar Abasaglar as biosimilar medicines requiring comprehensive comparability exercises (89).

Note: Implementation of automatic interchangeability designation would require either legislative amendment to PHS Act §351(k)(4), regulatory guidance interpreting existing statutory authority, or a combination of approaches. This analysis presents the scientific rationale; legal implementation pathways require further evaluation.

This divergence created not only practical challenges but also ethical disparities, with European patients gaining earlier access to affordable insulin biosimilars while American patients faced prolonged monopolistic pricing. The analytical and functional characterization required for European biosimilar approval differed substantially from the pharmaceutical quality and bioequivalence studies expected for US drug applications, forcing sponsors to generate redundant evidence packages that wasted resources without advancing scientific knowledge. Table 4 documents these historical divergences and their resolution, providing a template for NSPT harmonization.

**Table 4:** Regulatory convergence opportunities through NSPT classification.

Product category	US historical pathway	EU pathway	Current status	NSPT harmonization potential	Interchangeability implications
Insulin products	NDA/505(b)(2) pre-2020	Biosimilar	BLA post-2020 (7)	Not NSPT (receptor agonist)	Demonstrates the feasibility of transition
Somatropin	NDA/505(b)(2) (43)	Biosimilar	BLA eligible	Growth factor, not NSPT	Precedent for abbreviated pathways
TNF inhibitors	BLA/biosimilar	Biosimilar	Harmonized	Core NSPT class	Should receive automatic interchangeability
VEGF inhibitors	BLA/biosimilar	Biosimilar	Harmonized	Core NSPT class	Should receive automatic interchangeability
Golimumab	No US biosimilar	2 EU biosimilars (44)	Divergent	NSPT would enable harmonization	EU experience supports interchangeability
PCSK9 inhibitors	No biosimilars	No biosimilars	Void globally	NSPT would clarify requirements	Functional similarity ensures interchangeability

The March 2020 transition of insulin and other specified proteins from drug to biological product status in the United States represents a landmark demonstration that fundamental regulatory reform is both feasible and beneficial (13). The FDA successfully transitioned 39 approved insulin products from NDA to BLA status, enabling future insulin biosimilars to use the 351(k) approval pathway. This transition required careful coordination but proved that classification changes can be implemented without disrupting patient access—a critical precedent for NSPT implementation.

Notably, the NSPT framework proposes that biosimilars meeting analytical and functional similarity criteria could be considered for interchangeability designation, subject to statutory requirements. The current system requires additional switching studies and clinical data for interchangeability, representing a scientific anachronism for products with predictable, non-amplifying mechanisms. For NSPTs where therapeutic effect correlates directly with measurable functional activity, demonstrated analytical and functional similarity guarantees therapeutic equivalence regardless of switching patterns. Requiring additional clinical studies to illustrate what mechanism already ensures wastes resources, delays generic substitution, and maintains higher prices without scientific justification.

The ethical imperative for automatic NSPT interchangeability becomes clear when considering the patient impact of non-interchangeable biosimilars. Patients stable on reference products face insurance-mandated switching to non-interchangeable biosimilars, creating anxiety and potential nocebo effects. In contrast, those initiated on biosimilars may be denied access to reference products despite therapeutic equivalence. Physicians waste time navigating prior authorizations and appeals for clinically meaningless distinctions. Pharmacists cannot exercise professional judgment to substitute equivalent products. This bureaucratic complexity serves no scientific purpose for NSPTs, where functional equivalence ensures clinical equivalence.

International regulatory reliance mechanisms could accelerate global NSPT adoption. Many smaller regulatory agencies rely on assessments from stringent regulatory authorities when evaluating biosimilar applications. If FDA and EMA jointly adopted the NSPT classification, this standard would cascade through reliance pathways, creating global alignment. The World Health Organization’s prequalification program could explicitly recognize NSPT classification to promote access in resource-limited settings. A phased implementation roadmap would proceed as follows: Phase 1 (Year 1) would establish an FDA-EMA bilateral technical working group to draft joint scientific principles; Phase 2 (Years 1–2) would convene an ICH Q-series working party and pilot mutual recognition for select NSPT classes; Phase 3 (Years 2–3) would update WHO prequalification to recognize NSPT classification and engage emerging market regulators through ICMRA; Phase 4 (Years 3–5) would target full ICH guideline adoption with global convergence on evidence requirements. Table 1 incorporates these phases with specific milestones.

## 8 Implementation considerations for NSPT classification

The successful implementation of NSPT classification requires careful consideration of operational, scientific, and policy factors that could influence adoption and effectiveness. While the scientific rationale for mechanism-based classification is compelling, practical implementation must address stakeholder concerns, establish transparent governance processes, and provide sufficient flexibility to accommodate product-specific considerations while maintaining the standardization benefits that justify the classification system.

Implementing NSPT classification would require establishing transparent processes for determining product eligibility based on the mechanism of action. This determination should occur early in biosimilar development, ideally at the pre-IND meeting stage, to enable appropriate planning of the development program. Sponsors would submit a mechanistic rationale demonstrating that their product meets NSPT criteria, including evidence that the primary therapeutic effect operates through ligand neutralization, cascade blockade, or other non-signaling mechanisms. Regulatory agencies would need to establish review processes for these mechanistic assessments, involving specialized review teams with expertise in protein pharmacology and mechanism-of-action determination.

Standardization of functional assay requirements represents a critical implementation challenge that must balance consistency with product-specific needs. While the NSPT framework establishes general principles for functional characterization, specific assay panels must be tailored to each product and its mechanism. For anti-cytokine antibodies, this might include multiple cell-based neutralization assays using different responsive cell lines, whereas complement inhibitors would require hemolysis assays and markers of complement activation. Regulatory guidance should establish minimum assay requirements for standard NSPT mechanisms while maintaining flexibility for novel targets or unique product characteristics.

Determining similarity margins for functional assays requires careful scientific consideration and may require product-specific adjustments. Unlike pharmacokinetic bioequivalence, where 80–125% boundaries are universally applied, functional similarity margins may need to reflect the specific dose–response relationships and therapeutic windows of individual products. For products with steep dose–response curves and narrow therapeutic windows, tighter similarity margins might be appropriate. In contrast, products with flat dose–response relationships and wide safety margins could accommodate broader ranges. The NSPT framework should establish principles for setting these margins based on mechanistic understanding rather than arbitrary standards.

Quality system considerations for NSPT biosimilar development must ensure that streamlined clinical requirements do not compromise product quality or manufacturing consistency. The reduced emphasis on clinical studies places greater weight on analytical and functional characterization, requiring robust analytical method validation and strict adherence to quality standards. Manufacturing process controls become even more critical (93) when clinical studies are not available to detect subtle quality variations that might impact safety or efficacy. The NSPT framework should emphasize that abbreviated clinical development must be accompanied by enhanced analytical rigor and quality oversight.

Stakeholder engagement and education will be essential to the successful implementation of NSPT. Healthcare providers accustomed to seeing clinical trial data for biosimilars may initially question products approved primarily on analytical and functional evidence. Professional education programs should explain the scientific rationale for NSPT classification and demonstrate how functional assays provide more sensitive detection of meaningful differences than clinical trials. Patient advocacy groups should be engaged to understand and support mechanism-based events that could accelerate access to affordable biosimilars without compromising safety or efficacy standards.

Post-market surveillance strategies for NSPT biosimilars should be tailored to address any residual uncertainties that remain unresolved after pre-market assessment. With streamlined pre-approval clinical data, pharmacovigilance becomes the primary safety net for NSPT biosimilars. Robust post-approval monitoring should include risk-based pharmacovigilance protocols proportionate to residual uncertainty, real-world evidence collection through disease-specific registries and claims databases integrated with FDA Sentinel and EMA DARWIN-EU systems, mechanism-specific safety endpoints tailored to each NSPT class, and defined protocols for signal detection and response. The NSPT framework should establish clear expectations that abbreviated pre-market clinical development must be accompanied by enhanced post-market monitoring that can detect and respond to any unexpected safety signals in broader patient populations.

Implementation risks warrant careful consideration and proactive mitigation. Review inconsistency represents a potential challenge when regulatory decisions rely heavily on complex analytical data interpretation. Development of standardized analytical guidelines, reference datasets establishing similarity benchmarks for each NSPT mechanism class, and inter-laboratory validation programs would help ensure consistent regulatory interpretation across reviewers and jurisdictions. Equity considerations also merit attention: a shift toward analytically intensive development packages may favor larger manufacturers with advanced capabilities. Mitigation strategies could include enhanced pre-submission meeting programs to reduce technical uncertainty for smaller developers, public-private partnerships to develop standardized assay platforms, and leveraging contract research organization networks to democratize access to sophisticated analytical methods.

## 9 Future perspectives: expanding and refining NSPT classification

The initial implementation of NSPT classification should focus on well-characterized products with established mechanisms and extensive clinical experience, but the framework has potential for expansion as scientific understanding advances. Future refinements could incorporate emerging therapeutic modalities, address edge cases that challenge current boundaries, and evolve based on accumulated regulatory experience with mechanism-based classification. The dynamic nature of biological science requires that any classification system maintain sufficient flexibility to accommodate discoveries while preserving the standardization benefits that justify its existence.

Emerging therapeutic modalities may require expansion or modification of NSPT criteria as new mechanisms of action are discovered and validated. Bispecific antibodies that simultaneously neutralize two ligands clearly fit within the current NSPT framework, as their mechanism involves dual target sequestration without signal transduction. However, novel constructs that combine neutralization domains with other functional modules (94) may require careful evaluation to determine whether their composite mechanisms qualify for NSPT classification. The framework should establish processes for evaluating novel mechanisms as they emerge rather than attempting to anticipate all possible future developments.

Advances in analytical and functional characterization methods could enable the inclusion of products currently excluded from NSPT classification due to complexity or uncertainty. As understanding of structure–function relationships improves and analytical methods become more sophisticated, products with mechanisms that currently seem too complex for streamlined development may become amenable to NSPT approaches. For example, antibodies with complex effector functions that presently require clinical assessment might qualify for NSPT classification if functional assays can reliably predict their immunological effects.

The accumulation of regulatory experience with NSPT biosimilars will provide critical data for refining classification criteria and evidence requirements. Post-market surveillance data from biosimilars approved under NSPT principles will validate whether analytical and functional similarity reliably predicts clinical equivalence across diverse patient populations and clinical settings. Any unexpected differences identified in real-world use should trigger a reassessment of the evidence requirements for the affected product class. This iterative refinement process ensures that the NSPT framework evolves based on empirical evidence rather than theoretical assumptions.

International adoption of NSPT principles could drive global convergence in biosimilar regulation, creating opportunities for further harmonization and mutual recognition agreements. As regulatory agencies gain experience with mechanism-based classification, opportunities may emerge for collaborative reviews where agencies share assessments of analytical and functional data while conducting jurisdiction-specific evaluations of regional requirements. The NSPT framework could serve as a foundation for international standards that facilitate global biosimilar development while respecting sovereign regulatory authority.

The integration of artificial intelligence and machine learning approaches into biosimilar development could enhance NSPT implementation by improving the prediction of functional similarity from analytical data. Advanced computational models trained on accumulated biosimilar data could identify subtle structural features that correlate with functional differences, enabling more targeted analytical characterization. Machine learning algorithms could also optimize functional assay selection by identifying which assessments provide the most discriminatory power for specific product classes. These technological advances should be incorporated into the NSPT framework as they mature and demonstrate reliability.

## 10 Conclusions: recommendations for regulatory consideration

The accumulated evidence presented in this review establishes a substantial scientific, policy, and economic rationale for the FDA and EMA to evaluate the Non-Signaling Protein Therapeutics classification framework. The convergence of regulatory modernization initiatives, accumulated biosimilar experience, and patient access considerations creates a timely opportunity for regulatory agencies to assess whether mechanism-based classification merits formal implementation. This analysis suggests that regulatory agencies consider establishing NSPT classification guidelines and evaluating interchangeability considerations for qualifying biosimilars through appropriate statutory and regulatory processes.

The scientific foundation for NSPT classification is supported by substantial evidence. Forty-three currently licensed biological products demonstrate predictable, mechanism-based pharmacology, in which functional assays directly measure therapeutic activity without signal amplification or contextual complexity. The successful development of biosimilars for anti-TNF, anti-VEGF, and complement-inhibitor therapeutics, supported by analytical and functional evidence, suggests that clinical trials may provide limited additional discriminatory information for these mechanisms. The FDA’s November 2025 draft guidance, acknowledging that comparative efficacy studies should address specific uncertainties rather than serve as defaults, provides a regulatory framework for considering NSPT implementation (2).

The resource allocation and access considerations supporting NSPT classification warrant attention. Extended development timelines associated with current evidence requirements may delay biosimilar availability and sustain limited market competition. The products identified in Table 3, including secukinumab, evolocumab, and omalizumab, represent cases in which limited biosimilar development, despite expired patents, may reflect regulatory uncertainty that mechanism-based classification could address. Careful evaluation of whether current evidence requirements are optimally calibrated to scientific uncertainty may identify opportunities to better align regulatory practice with resource stewardship principles.

We propose specific actions for immediate implementation:

- For FDA and EMA leadership: Issue joint guidance establishing NSPT classification with clear inclusion criteria based on mechanism (ligand neutralization, complement blockade, non-signaling interception) and standardized evidence requirements prioritizing analytical and functional characterization. Set a target of March 2026 for draft guidance and December 2026 for final implementation.
- For automatic interchangeability: Recognize that NSPTs meeting analytical and functional similarity criteria are therapeutically equivalent to their mechanisms. Eliminate additional switching studies and clinical requirements that lack a scientific basis and impede pharmacy-level substitution. This single reform could save billions in healthcare costs while improving patient access.
- For global harmonization: Convene an International Conference on Harmonisation working group specifically focused on NSPT classification to establish worldwide standards. Engage WHO, regulatory authorities from emerging markets, and patient advocacy groups to ensure broad stakeholder alignment. Target completion of harmonized guidelines by 2027.
- For transparency in implementation, establish public databases to track NSPT biosimilar applications, review timelines, and record approval decisions, ensuring accountability and identifying remaining barriers. Publish quarterly reports on biosimilar approvals under NSPT pathways versus traditional requirements to demonstrate impact.
- For continuous improvement, create standing advisory committees comprising academic experts, industry scientists, and patient advocates to refine NSPT criteria based on accumulated experience. Establish mechanisms for petitioning to add new products to the NSPT classification as mechanisms are validated.

The pharmaceutical industry must also embrace responsibility for advancing NSPT implementation. Originator companies should recognize that perpetuating monopolies through regulatory uncertainty undermines long-term industry credibility and social license. Biosimilar developers should collaborate to generate shared functional assay standards and validation protocols that strengthen scientific confidence in abbreviated pathways. Both segments benefit from precise, predictable regulatory requirements that reward genuine innovation while enabling competition for mature products.

Healthcare systems and payers should actively advocate for NSPT classification as essential for sustainability. The projected $232 billion in biological product spending approaching patent expiration represents an existential challenge for healthcare financing that only robust biosimilar competition can address (4). Payers should explicitly support automatic interchangeability for NSPT biosimilars to maximize substitution rates and cost savings.

Patient advocacy organizations must recognize NSPT classification as a fundamental access issue. The current system that maintains monopolistic pricing through regulatory barriers rather than patent protection denies patients life-changing therapies based on ability to pay rather than medical need. Advocates should demand that regulatory agencies prioritize patient access over bureaucratic caution by implementing the NSPT classification immediately.

This analysis suggests that current regulatory approaches for NSPTs may benefit from reconsideration. The NSPT framework provides a scientifically grounded approach that maintains safety and efficacy standards while potentially streamlining unnecessary requirements. Further regulatory evaluation would determine whether this framework advances public health objectives.

The evidence is clear: the FDA and EMA should demonstrate that patient welfare, scientific integrity, and healthcare sustainability guide their decisions by establishing NSPT classification with automatic interchangeability through established regulatory processes. The millions of patients awaiting affordable biological therapies deserve nothing less than proactive regulatory leadership that balances patient needs with regulatory rigor. Further evaluation of this framework is warranted.
